# Relationship between orthorexia nervosa and self-esteem in Tunisian medical students

**DOI:** 10.1192/j.eurpsy.2023.1804

**Published:** 2023-07-19

**Authors:** M. Abdelkefi, R. Masmoudi, R. Jbir, F. Cherif, I. Feki, R. Sellami, J. Masmoudi

**Affiliations:** psychiatry A, Hedi Chaker university hospital, sfax, Tunisia

## Abstract

**Introduction:**

The effect of self-esteem in eating disorders has been investigated in several studies, but it’s still not extensively investigated in orthorexia nervosa.

**Objectives:**

To study the prevalence and factors associated with orthorexic eating behaviors in medical students and it’s relation with self-esteem.

**Methods:**

A cross-sectional study was conducted through an online survey among medical students of the faculty of medicine of Sfax (Tunisia). Participants completed an anonymous self-administered questionnaire. We collected their sociodemographic and clinical data. Orthorexia nervosa (ON) was assessed using the self-reported scale, ORTO-15. We used the Rosenberg’s self-esteem scale to assess self-esteem.

**Results:**

Ninety five medical students completed the survey. The mean age was 25.8±3.4 years and the sex ratio (F/M) was 3.75. The average body mass index was 23.64±3.53 kg /m2.

Fifty-eight percent of the students (58%) reported that they were dissatisfied with their eating habits, and 27.4% tried to control their weight. Several methods of weight control were used, the most frequent (65.4%) were diet and physical exercise, none resorted to laxatives and 8.4% consulted a nutritionist.

Self-esteem was very low in 27.1% and low in 34.7% of the students.

Overall, the prevalence of orthorexia among our participants was 52.6%. The mean score of the ORTO-15 was 39.19±4.48.

Orthorexia was significantly correlated with the use of weight control measures (p=0.035) and physical activity (p=0.042).

Students with low self-esteem had higher tendency for orthorexia but with no significant correlation.

**Conclusions:**

Our study supports a non-negligible frequency of orthorexic behaviors in medical students but future studies are needed to assess the direct effect of self-esteem on orthorexia.

**Disclosure of Interest:**

None Declared

